# Anti-tumor potential of Harmine and its derivatives: recent trends and advancements

**DOI:** 10.1007/s12672-025-01893-w

**Published:** 2025-02-15

**Authors:** Hemant Joshi, Sakshi Bhushan, Tanisha Dimri, Deepak Sharma, Katrin Sak, Abhishek Chauhan, Ritu Chauhan, Shafiul Haque, Faraz Ahmad, Manoj Kumar, Hardeep Singh Tuli, Damandeep Kaur

**Affiliations:** 1 School of Biotechnology, Jawaharlal Nehru University, New Delhi, 110067 India; 2 Department of Plant Science, University of Manitoba, Winnipeg, MB R3T 2N2 Canada; 3 Department of Biotechnology, All India Institute of Medical Science, New Delhi, 110029 India; 4 NGO Praeventio, 50407 Tartu, Estonia; 5 Amity Institute of Environmental Toxicology Safety and Management, Amity University, Noida, Uttar Pradesh 201313 India; 6 Department of Biotechnology, Graphic Era Deemed to Be University, Dehradun, Uttarakhand 248002 India; 7 Research and Scientific Studies Unit, College of Nursing and Health Sciences, Jazan University, 45142 Jazan, Saudi Arabia; 8 Department of Biotechnology, School of Bio-Sciences and Technology (SBST), Vellore Institute of Technology, Vellore, 632014 India; 9 Department of Chemistry, Maharishi Markandeshwar University Sadopur, Ambala, 134007 India; 10 Department of Bio-Sciences and Technology, Maharishi Markandeshwar Engineering College, Maharishi Markandeshwar (Deemed to Be University), Ambala, Mullana 133207 India; 11 University Center for Research and Development (UCRD), Chandigarh University, Gharuan, Mohali, Punjab India; 12 School of Medicine, Universidad Espiritu Santo, Samborondon, Ecuador

**Keywords:** Harmine, Alkaloid, Anti-cancer, Natural product, Cancer therapeutics

## Abstract

Harmine is a β-carboline alkaloid derived from *Peganum harmala*, showing a solid antitumor potential in different types of human cancer cells. Unfortunately, the clinical application of this natural alkaloid has been impeded till now by severe toxic side effects, especially neurotoxicity, besides its poor water solubility. Therefore, over the recent years, several semisynthetic derivatives of harmine have been prepared and studied concerning their abilities to inhibit tumor cell proliferation, survival, angiogenesis, migration, and invasion in diverse preclinical models. This review article summarizes the anticancer effects of harmine and its synthetic derivatives, demonstrating their high potential to be developed as novel anticancer drugs to supplement our current therapeutic arsenal in the fight against the globally increasing rate of malignant disorders.

## Introduction

Historically, more than 60% of all approved anticancer drugs have been derived from different natural sources [1], suggesting that nature can provide promising lead structures for developing oncological medicines. This is especially important considering the continuously increasing incidence rate of malignant disorders all over the world [2]. Therefore, identifying and characterizing novel natural compounds with diverse anticancer properties is critical to improving the clinical outcome and quality of life of patients in the future [3–6].

Harmine is a naturally occurring β-carboline alkaloid that was initially derived from wild rue (*Peganum harmala*) [7, 8]. Several recent studies have shown that this natural compound can exert multiple anticancer activities by inhibiting various tumoral cells' proliferation, cell cycle progression, survival, angiogenesis, and metastatic ability [7, 9]. In doing so, harmine can interact with many different cellular target sites and modulate the downstream signaling pathways in diverse cancer cells, including colon cancer. [10], breast cancer [11, 12], ovarian cancer [13] and prostate cancer [14]. This agent can also display anti-inflammatory effects, alleviating the inflammatory tumor microenvironment and impeding the malignant progression [7]. Moreover, several investigations have shown the ability of harmine to decrease the resistance of cancer cells to conventional chemotherapeutic drugs, resulting in enhanced therapeutic responses and allowing them to reduce their efficient doses [7, 15]. All these preclinical results highlight harmine as an attractive lead compound for further drug development. However, its clinical use has still been limited by low solubility and several toxic side effects that have led scientists to produce different semisynthetic derivatives [7].

**Fig. 1:**
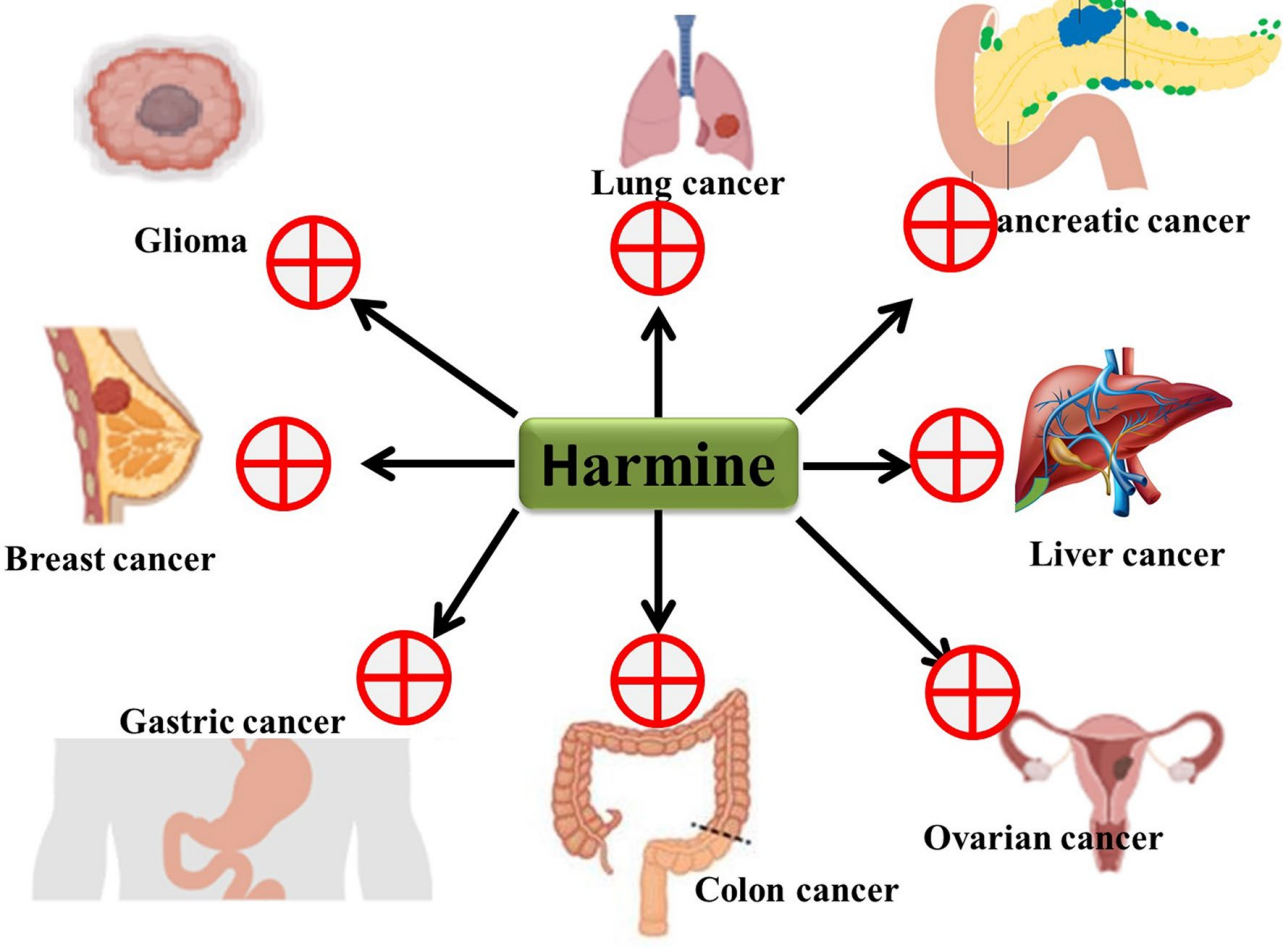
Schematic representation of the chemopreventive potential of harmine by restricting the cancerous growth in different types of cancer

In this review article, all the recent preclinical results on the anticancer activities of harmine and its synthetic derivatives are compiled to provide a comprehensive and contemporary picture of the current status of potential clinical applications of this natural compound in future anticancer therapies (Fig. 1). Further work is required to get more information about these valuable insights and investigate the detailed molecular mechanisms underlying these actions.

## Chemistry/structure–activity relationship of harmine and its derivatives

**Fig. 2:**
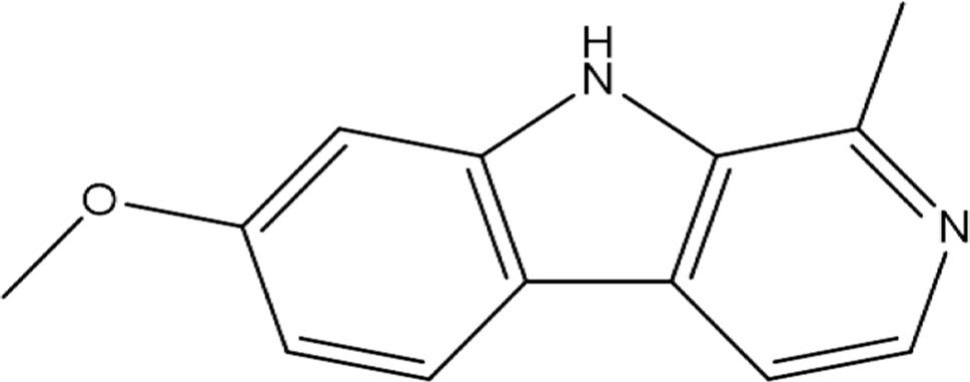
Chemical structure of 7-methoxy-1-methyl-9*H*-pyrido[3,4*-b*]indole (Harmine)

7-methoxy-1-methyl-9H-pyrido[3,4-b]-indole (Fig. 2) is the chemical formula for harmine. This naturally occurring β-carboline alkaloid has been shown to have a wide range of biological and pharmacological properties, including anti-inflammatory, anti-cancer, anti-microbial, anti-oxidant, anti-depressant, and neuroprotective properties. Harmine and its derivatives possess potential pharmacological relevance among all the β-carboline groups [16]. The plant *P. harmala* is a robust herbal remedy because of its abortifacient, emmenagogue, hallucinogenic, lactagogue, and hypothermic qualities. People in Northwest China have used *P. harmala* seed extracts for centuries to treat intestinal tract cancer and malaria. Perennial herbaceous *P. harmala* was formerly classified as a part of the Zygophyllaceae family, but more recently, it was determined to belong to the Nitrariaceae family. In addition to *P. harmala*, harmine was found in *P. multisectum* and *P. nigellastrum*. Additionally, Harmine was separated from *Andrographis paniculata's* leaves and roots. Traditional Chinese medicine helps treat respiratory diseases, dysentery, indigestion, influenza, and snakebite. Furthermore, it has been discovered that *Oxalis tuberosa* roots and *Bantisteriopsis caapi* vine stalks are excellent sources of harmine. Harmine was shown to be present in considerable quantities in *P. caerulea* and minor quantities in *P.incarnata*, according to a quantitative examination [17].

**Fig. 3:**
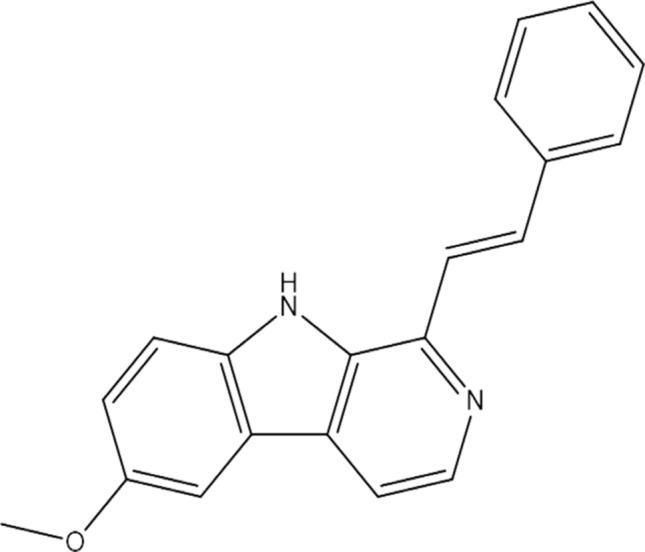
Chemical structure of harmine derivative *E*-6-methoxy-1-styryl-9*H*-pyrido[3,4-*b*]indole (JKA97)

Methanol can extract harmine, which can then be isolated using column chromatography techniques such as silica gel [18]. Researchers successfully synthesize the derivatives of harmine chemically (E)-6-methoxy-1-styryl-9H-pyrido-[3,4-b]-indole (Fig. 3), followed by HPLC–MS analysis [19]. The time-dependent density functional theory (TD-DFT) investigation established the structure–activity relationship (SAR) of harmine derivatives and demonstrated the critical roles that position 3 and 9 played in the anticancer and neurotoxic properties [20]. The SAR analysis results showed that replacing the proton at position 3 with a long alkyl or aryl group decreased the anticancer activity. Similarly, replacing the proton at position 9 with a short alkyl or aryl group increases the cytotoxicity [21]. According to SAR analysis, harmine derivatives that were N9-alkylated have shown distinct cytotoxic effects, whereas N2-alkylated showed improved cytotoxic activities. The alkoxy chain length also affected cytotoxicity and cell line specificity [22]. Harmine's 7-methoxy was crucial in determining its neurotoxic effects, and replacing the methoxy at position 7 with a large alkoxy group eliminated the neurotoxic effects. JKA97 exhibited more anticancer activity than harmine against MCF7, MCF7p53kD, and MDA-MB-468 cell lines. Furthermore, in vitro and in vivo data showed that harmine substitutions at positions 2, 7, and 9 resulted in a notable decrease in side effects and an excellent enhancement of antitumor activity.

## Anti-cancerous mechanisms of harmine and its derivatives

### Apoptosis induction

**Fig. 4:**
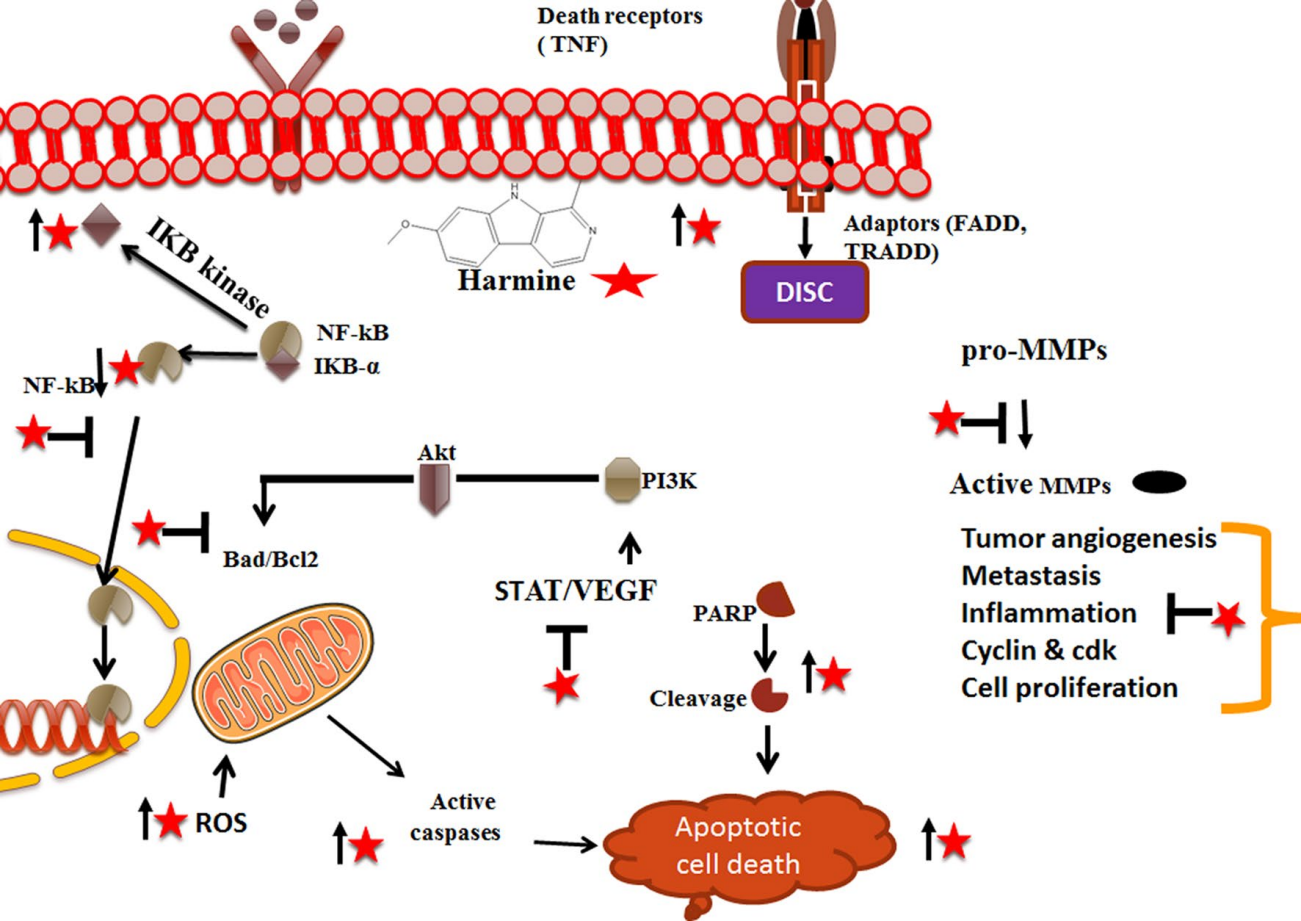
Mechanistic insights of chemopreventive potential of harmine by modulating different proteins involved in various pathways such as angiogenesis, metastasis, inflammation, cell cycle arrest, and cell proliferation

Apoptosis or Programmed Cell Death (PCD) is a natural process of eliminating old cells from the human body. It is a well-known fact that anticancer cells rely on the stimulation of apoptosis to remove malignant cells effectively. Interestingly, regulation and deregulation of signaling pathways in cancer play a crucial role in managing abrupt and uncontrolled cellular proliferation [23]. The apoptotic pathway of harmine action can be divided into extrinsic (death receptor) and intrinsic (mitochondrial) pathways. The extrinsic mode of action involves successive activation of caspase 8 and caspase 3 (Table 1). In contrast, the inherent pathway consists of activating endogenous stimuli, leading to the release of cytochrome c and the activation of caspase 9, which activates caspase 3 (Fig. 4). It was observed that B16F10 cells treated with harmine showed nuclear fragmentation, apoptotic bodies, and DNA ladder formation, indicating the presence of an apoptotic phenomenon in action. Moreover, harmine treatment resulted in a reduction of Bcl-2 and enhancement of Bax, caspase-3, 9, 8, and increased Bid efficiently [24, 25]. It also induced apoptosis in conditions such as liver cancer [26], thyroid cancer [27], and neuroblastoma [28] by regulating the Bcl-2/Bax ratio using mitochondrial pathways, respectively. Interestingly, harmine also showed its effect on breast cancer by efficiently upregulating Bax and downregulating Bcl-2, p-AKT, p-mTOR, and p-Erk [28].

**Table 1:** An overview of the anti-proliferative effect of harmine against different cancers

S. no.	Type of cancer	Subjective model	Physiological effect	Mechanism of action	References
1	Breast cancer	MDA-MB-231 and MCF-7, mouse model	Apoptosis induction and Anti-proliferative effect	↓TAZ, ↓p-Erk, ↓p-Akt and ↓Bcl-2, ↑Bax	[11, 12, 37]
2	Bladder cancer	RT112, RT4, SW780, BIU87, and 5637	Apoptosis induction and Anti-angiogenesis	↓VEGFR2, ↓PARP, ↓caspase, ↓p-AKT and p-ERK1/2	[38]
3	Glioblastoma	U-87 MG cells	Anti-proliferative and Antimetastasis	↓MMP-3	[32, 39–41]
4	Ovarian cancer	SKOV-3 cells	Antiproliferative effect	↓ERK1/2, ↓CREB, ↓VEGF, ↓MMP-2, and MMP-9	[42]
5	Gastric cancer	BGC-823, SGC-7901 cell lines	Apoptosis induction and Anti-proliferative effect	↓COX-2, ↓PCNA, ↓Bcl-2 and MMP-2, ↑Bax	[34, 43–46]
6	Thyroid Cancer	TPC-1 cell line	Apoptosis induction and Anti-proliferative effect	↓Bcl-2/Bax, ↑caspase-3	[27, 47]
7	Neuroblastoma	SKNBE, KELLY, SKNAS, SKNFI cells	Apoptosis induction	↑caspase-3/7 and caspase-9, ↓DYRK2	[28]
8	Esophageal squamous cell carcinoma	ESCC cells	Apoptosis induction, Cell cycle arrest, Anti metastasis, Anti-proliferative effect	↓CHOP-mediated sestrin-2, ↓AMP-protein kinase B	[29]
9	Colorectal carcinoma	SW620 cells	Apoptosis induction, Cell cycle arrest, Anti-proliferative effect	↓cyclin D1, ↑cyclin A, ↑E2 and B1, ↑CDK1/cdc2, ↑Myt-1 and p-cdc2 (Tyr15), ↓Bcl-2, ↓Mcl-1, ↓Bcl-xL, ↑Bax, p-↓ERK, ↓p-Akt (Ser473) and p-Akt (Thr308)	[48]
10	Leukaemia	NB4 cells	Cell cycle arrest and Anti-proliferative effect	↓DNMT1, ↓p15	[49]

Additionally, harmine and its derivatives have also been observed for their participation in tumor cell apoptosis/autophagy via regulation of other critical mechanisms, including pro-inflammatory cytokines such as tumor necrosis factor-α, interleukin (IL)-1β, IL-6, and macrophages [9, 15, 24]. Recently, Fan et al., 2023 reported the role of harmine in inducing endoplasmic reticulum stress, which in turn suppresses esophageal squamous cell carcinoma via expression of CHOP-mediated sestrin-2 which further contributes to autophagosome formation [29]. Several studies have indicated that harmine and about nine derivatives of harmine are responsible for efficient anti-cancer effects in liver and lung cancers [30]. Since such conditions involve overexpression of tyrosine phosphorylation and regulation of kinase 1A (DYRK1A) during uncontrolled cell proliferation and tumorigenesis, harmine has been reported as an effective inhibitor of DYRK1A [31]. Furthermore, harmine hydrochloride, a derivative of harmine, has been well established for its anticancer potential via regulating exaggerated levels of p21 and Bax and reduced levels of Bcl-2 and Bcl-xl, respectively [32]. Another harmine derivative (B-9–3) that was synthesized selectively induced anticancer response by inducing apoptosis of endothelial cells via efficient disruption of VEGF-A/VEGFR2 response [33].

In cancer cell lines, SGC-7901 and MGC-803, harmine was able to induce apoptosis through regulation of mitochondrial-mediated Akt/mTOR/p70S6K pathway and autophagy (adenosine 5’-monophosphate-activated protein kinase) pathway which is in turn linked to its ability to modulate cell microtubule protein light chain 3 (LC3-II), Beclin-1, and P62. It was observed that harmine showed its anti-cancer activity by upregulating apoptosis-related factors/proteins [34]. Shen et al., 2018, reported that harmine plays a critical role in cell cycle arrest and apoptosis in NSCLC via activating reversion-inducing cysteine-rich protein with Kazal motif expression and its downstream signaling, leading to the downregulation of MMP-9 and E-cadherin [35]. Interestingly, harmine attenuated the expression of Mcl-1 in addition to sensitizing NSCLC cells to Bcl-2 inhibitors through DYRK1A inhibition, inducing apoptotic death of cells [36].

The introduction of an appropriate substituent (at position 9) increased the affinity of harmine to DNA, resulting in strong topoisomerase I inhibitory effects [50]. Since Cyclin-dependent kinases (CDKs) play a vital role in the regulation of DNA replication and S phase progression, harmine and many of its derivatives have been reported to inhibit Cdk1/cyclin B, Cdk2/cyclin A, and Cdk5/p25, resulting in antiproliferation of defective cells [51]. Moreover, DYRKs are essential in regulating the cell cycle and survival [52]. Harmine, along with many harmine derivatives, was synthesized by substitution at positions 2, 7, and 9 and, in turn, showed inhibitory effects against DYRK1A [53]. Haspin (haploid germ cell-specific nuclear protein kinase) overexpression/deletion leads to defective mitosis, and inhibition of haspin has potent antitumor effects [54]. Interestingly, harmine and its derivatives have been reported to be a potent inhibitor of haspin, thus indicating their efficient role in cell cycle arrest [55]. Similarly, harmine resulted in the inhibition of proliferating HepG2 cells by reducing the proportion of cells in the G_0_/G_1_ phase and increasing the S and G_2_/M proportion in a dose-dependent manner by activation of caspase 3 and caspase 9, played a role in the downregulation of Bcl-2, Mcl-1, and Bcl-xl expression [26]. Additionally, numerous reports have established the anti-cancer/anti-tumor effects of harmine and its synthesized derivatives in different cancer types. Therefore, the meticulous effect of harmine and its derivatives as a therapeutic candidate in cancerous conditions/disease can be attributed to the synergistic impacts of their tendency to interfere with DNA, CDKs inhibition, regulation of DYRKs, and haspin and promotion of angiogenesis [15].

### Anti-metastasis and anti-angiogenesis

**Fig. 5:**
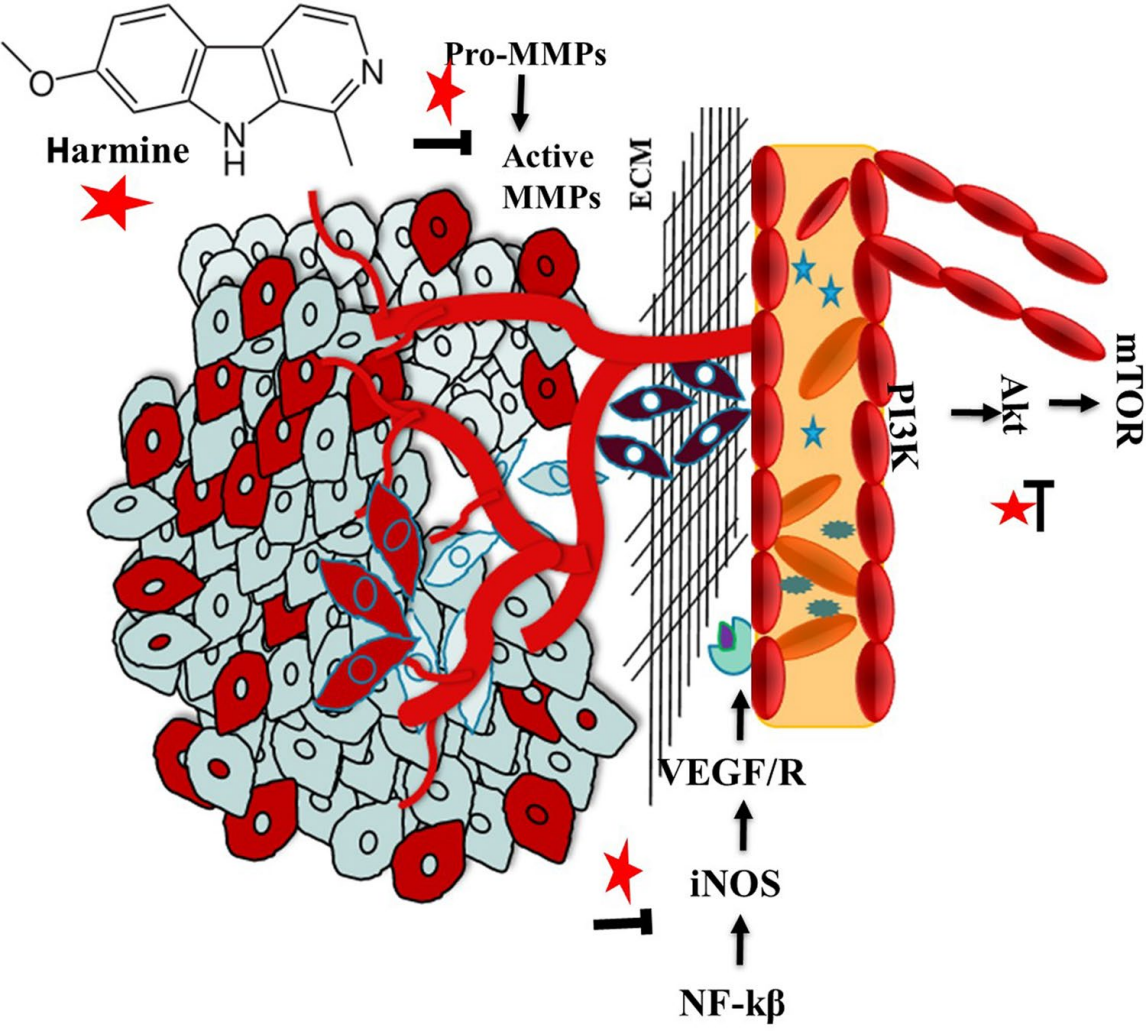
Mechanistic representation of anti-angiogenic and anti-metastatic effects of harmine

It is a well-known fact that metastasis is an essential process in cancer progression. Harmine and its derivatives have recently gained attention for their potential to target various types of cancer (Table 1). Their mode of action depends upon inhibiting migration and invasion of cancer cells through the downregulation of matrix metalloproteinases (MMPs) and enzymes involving angiogenesis in the extracellular matrix (Fig. 5). Several studies have reported the tendency of harmine to reduce the expression of MMP-3 and MMP-9, which resulted in the attenuation of the adverse effect of malignant cells [10, 39, 42, 43]. In addition, Zhu et al. (2021) observed that harmine significantly suppressed the migration of U251-MG cells by inhibiting the expression of MMP-2, MMP-9, and VEGF (Fig. 5) [56]. Harmine derivative B-9–3 has shown a significant angiogenic and antitumor action against Lewis lung cancerous cells (LLC) by inhibiting the growth of vascular fibroblasts and endothelial cells, which in turn stimulated the process of regression in LLC, ovary (SKOV-3) and prostate (22RV1) tumor cells respectively. Furthermore, the harmine derivative exaggerated the apoptotic rate by disrupting the VEGF-A/VEGFR2 pathway significantly [33]. Interestingly, harmine and its derivatives also regulated crucial cell signalling pathways in metastasis. Harmine can suppress multiple malignant phenotypes and inhibit the PI3K signaling pathway, thus regulating cell survival and motility, further enhancing their antimetastatic property [11, 47]. Vascular endothelial growth factor (VEGF) and its receptor (VEGFR) are the leading candidates in angiogenesis. Harmine and its analogs have shown the ability to downregulate the VEGF expression and VEGFR activation, thus affecting/interrupting angiogenic signaling through cells [7, 38]. Harmine downregulates VEGF expression and inhibits VEGFR activation, disrupting angiogenic signaling. This suppression prevents the formation of new blood vessels necessary for tumor sustenance and growth (Fig. 5). Moreover, harmine directly impairs the proliferation and migration of endothelial cells, essential for angiogenesis. Studies have shown that harmine reduces endothelial cell viability and migration, disrupting angiogenesis in a dose-dependent manner by activating p53 in endothelial cells [38, 57]. This effect is crucial for hindering the vascularization of tumors. Nafie et al., reported significant inhibition of migration and invasion of breast cancer cells in humans via proteasome-dependent Twist1 degradation [58].

### Anti-inflammatory

Harmine and its derivatives have potent anti-inflammatory effects, which work differently. Zheng et al. [59] demonstrated that the molecular targets influence the mode of inflammatory response. These β-carboline alkaloids, prominently found in the seeds and roots of *P. harmala*, have modulated inflammatory reactions at the molecular level. Some essential inflammatory pathways are affected via interactions of harmine, etc., with them. They particularly hinder significant pro-inflammatory cytokine secretions like TNF-α, IL-1β, and IL-6. NF-κB gets inactivated due to this blockage, thus leading to transcription regulation and reducing the inflammation process activation. The MAPK signaling pathway becomes another course modified by these substances so that mainly p38 MAPK and JNK pathways, which mediate inflammations, can be suppressed for harmine’s decline in their production levels [60].

Furthermore, harmine stops producing nitric oxide (NO) and prostaglandin E2 (PGE2), mediators for inflammation. In a study by Aslam et al. (2014), it was observed that down-regulation of inducible nitric oxide synthase (iNOS) and cyclooxygenase-2 (COX-2) expressions mitigated the inflammatory response [61]. The anti-inflammatory potential of harmine derivatives was assessed based on their ability to inhibit (5-LOX) enzyme activity. Harmine 1 and derivatives 2 and 3a–d demonstrated low activity against the 5-LOX enzyme, with IC_50_ values greater than 100 µM [16]. Harmine and other *P. harmala* alkaloids effectively block myeloperoxidase (MPO) activity, thus preventing MPO-mediated LDL oxidation, having a remarkable IC_50_ value of 0.26 µM. Additionally, molecular docking studies show that these compounds are highly selective for the active site of MPO. Harmine also inhibits the release of TNF-α, IL-6, and nitric oxide (NO) in LPS-induced Raw264.7 macrophages and THP-1 human cells in a dose-dependent manner [62].

## Synergistic effects of harmine and its derivatives

The majority of cancer patients experience treatment resistance, which makes it challenging to manage those who want to achieve better clinical outcomes. It has been demonstrated that harmine has strong synergistic effects when used with other anti-tumor medicines [63]. In recent years, harmine and its derivatives have been established as potent drug candidates for the treatment of cancer and tumorigenic conditions when coupled with chemotherapy medications and drugs. It was found that harmine has shown the ability to treat malignancies when administered in combination with paclitaxel. The combined dose of harmine and paclitaxel resulted in a synergistic anti-cancer effect that, in turn, downregulated matrix metallopeptidase 9 (MMP-9) and cyclooxygenase 2 (COX-2) in the gastric cancer cell lines, reducing proliferation and leading to apoptosis [64]. It was observed that cells administered with 2 ng/mL paclitaxel (2 ng/mL), harmine (4 ng/mL), or in combination attenuated COX-2 expression when compared to the effects of each medication alone [57]. Cancer cell death is significantly increased by this synergy, decreasing homologous repair and increasing DNA double-strand breaks. Therefore, the harmine radiation is propounded against pancreatic cancer radiotherapy [65]. Harmine upregulates TAP1, Tapasin, and Lmp2 in melanoma cells to improve MHC-I antigen presentation. ACB1801 decreases tumor weight and growth in melanoma-bearing mice, and when combined with anti-PD1 therapy, it dramatically enhances therapeutic results. In patients with melanoma, higher MHC-I expression is associated with improved survival and greater recruitment of CD8^+^ T cells [66].

Moreover, harmine also efficiently enhanced the cytotoxicity of gemcitabine against pancreatic cancer cell lines and induced apoptotic response. Since the AKT/mTOR pathway is a key mechanism for gemcitabine resistance in pancreatic cancer cells, this study demonstrated that harmine and gemcitabine, in combination, significantly suppressed the AKT/mTOR signaling pathway. Interestingly, it has also been observed that harmine acted as a sensitizer of docetaxel, which enhanced the inhibition against breast cancer cells [67]. Combination treatment of harmine with an LY294002, a specific inhibitor of PI3K/Ak, exaggerated cytotoxicity against gastric cancer cells [32]. In addition to this, harmine has been reported to counteract resistance to regorafenib in tumor cells via the AKT pathway through the inhibition of DYRK1A activity [13]. During this response, harmine in combination with regorafenib, efficiently inhibited HepG2 and Hep3B cancer cells via promoting apoptosis and upregulation of p-AKT when compared to single-drug administration.

Harmine increases sensitivity to anti-cancer medications by reversing TRIB2-mediated alterations in gene expression. It enables FOXOs to translocate to the nucleus, thus magnifying the cell death caused by BEZ235 in cancer cells. It's now well known that harmine may be the best option to defeat TRIB2-based therapy resistance, providing an effective way to enhance clinical outcomes for cancer patients [68]. Combining temozolomide and harmine synergistically enhances anti-tumor effects in glioblastoma multiforme (GBM) cells. This is due to the synergy effect of tumor suppressants that downregulate metalloproteinases required for cancer cell invasion. This, in turn, implies harmine as a potential agent that can ultimately improve the treatment outcomes for GBM [69].

Interestingly, harmine also showed the property of being a potential radiosensitizer. Lan et al. (2022) observed that a combination of radiotherapy and harmine enhanced the frequency of double-stranded breaks of DNA and further impaired homologous recombination in pancreatic cancer cells. This response, in turn, promoted the death of cancer cells, thus providing a new strategy to overcome tolerance in pancreatic cancerous conditions [60]. Harmine and its derivatives have shown efficacy as therapeutic candidates against melanoma in mice models via attenuating immune response and synergizing immunotherapy drugs. It was reported that in combination with anti-PD1/PD-L1 therapy, harmine resulted in improvement against cancer response in patients by upregulating the presentation of harmine major histocompatibility complex class I (MHCI) dependent antigens respectively [70].

A literature survey has revealed that harmine has been efficiently used in traditional herbal medicines and possesses excellent antitumor ability as it can effectively induce apoptosis and plays a crucial role in proliferation inhibition [5]. Harmine and its derivatives have shown crucial properties to reduce drug resistance when used along with other chemotherapeutic drugs. Interestingly, many more derivatives with lower toxicities and anticancer activities have been developed through modifying harmine structure. As evident from the above-mentioned reports and observations harmine has efficiently established itself as a promising therapeutic agent when considered in chemotherapy and radiotherapy. This approach can further be explored in cancer chemoprevention during clinical treatments and other applications. In conclusion, the mechanism of action of harmine and its derivatives in eradicating tumorigenic conditions is further needed to improve drug activity research and clinical translation.

## Safety aspects

Harmine encounters challenges in its application due to inadequate solubility and toxic side effects linked to high doses and extended exposure, which impede its clinical utility despite its notable antitumor efficacy. It is reported to display an IC_50_ value of ~ 100 nM, stimulating beta cell mitogenesis, presenting potent anticancer activities through apoptosis and autophagy, and showing anti-inflammatory effects, possibly through inhibiting the NF-κB signaling pathway [34, 71–74]. Research findings indicate that the LD_50_ of harmine is 26.9 mg/kg, leading to common signs of neurological toxicity in mice, such as convulsions, systemic muscle tremors, and even opisthotonos [75]. A preliminary exploration of the neurotoxic mechanism of harmine in *C. elegans* exposed a dose-dependent adverse impact on the nematodes' growth, development, and locomotion behavior. Even though the neurotoxicity induced by harmine does not present observable structural alterations in neurites or neuronal demise, it likely functions by obstructing acetylcholinesterase activity, resulting in excessive acetylcholine buildup, thereby influencing neural function [76]. Harmine can also induce acute cardiovascular toxicity, giving rise to alterations in ECG waves, heightened myocardial enzymes like lactate dehydrogenase, creatine kinase, and creatine kinase-MB, as well as a reduction in blood pressure and heart rate [77, 78]. Initially pinpointed as a selective inhibitor of dual-specificity tyrosine phosphorylation-regulated kinase 1a (DYRK1A) in glioma cells [71, 79], harmine's therapeutic efficacy is compromised by its off-target inhibitory effect on monoamine oxidase-A (MAO-A), inducing degradation and reuptake of monoamines such as serotonin and norepinephrine, consequently impacting various targets within the nervous system [80, 81]. Moreover, chronic harmine treatment also resulted in substantial weight loss, locomotion impairments, and transient tremors in socially defeated rats [82].

The enhancement of harmine's antitumor activity could be achieved through structural modifications and alteration in medication dosage, simultaneously transforming it into a less toxic compound having a vital clinical and translational impact. Harmine’s neurotoxicity has been a critical barrier to its clinical application, but emerging strategies offer promising avenues to address this issue. Among these, structural modifications, such as *N9-alkylation*, have shown potential in reducing the neurotoxic effects of harmine while retaining its therapeutic properties. For instance, modifications at the N9 position can alter the compound's pharmacokinetic profile, reducing its interaction with neuronal pathways associated with toxicity [30]. Modifications at the substantial hydrophobic groups situated at positions 2 and 9 enhance the antitumor efficacy of harmine, while the insertion of long-chain substituents at position 7 can diminish its neurotoxicity [83]. Replacing the 7-methoxy group with a bulky alkoxy moiety notably reduced neurotoxic impacts in mice [26]. Two harmine derivatives, 2DG-Har-01 and MET-Har-02, were developed by making modifications at the 2nd, 7th, and 9th positions of the harmine ring, endowing them with lower systemic toxicity and heightened therapeutic anti-cancer effects [64].

Encapsulation techniques, such as liposomal delivery systems and nanoparticles, are another promising approach. These methods can enhance harmine’s targeted delivery to diseased tissues, minimizing off-target effects and reducing exposure to the central nervous system, where neurotoxicity is most pronounced [67]. Preclinical studies suggest that these delivery systems not only improve safety but also enhance harmine’s bioavailability and therapeutic efficacy [70]. Our immediate focus is on refining these preclinical strategies. Structural modifications, such as *N9-alkylation*, are being optimized to achieve a balance between therapeutic activity and safety. Concurrently, encapsulation approaches are being developed to ensure precision targeting and controlled drug release, both of which are critical for minimizing adverse effects.

While preclinical modifications remain a priority, we recognize the importance of exploring clinical toxicity management strategies in the future. For example, combining harmine with neuroprotective agents may mitigate its toxic effects during therapy. Similarly, dose-sparing regimens or adjunctive therapies could offer practical solutions in a clinical setting, as has been demonstrated with other neurotoxic agents [84]. A recent study synthesized harmine encapsulated in polylactic-co-glycolic acid (PLGA) nanoparticles (Ha-PLGA-NPs) with an IC_50_ value of 87.74 µg/mL, indicating a favorable selectivity for cytotoxicity towards cancer cells as compared to human foreskin fibroblasts and concentration-dependent inhibition of angiogenesis [85]. The derivatives of harmine-nitric acid donors showcased decreased acute toxicity, beneficial plasma stability, and remarkably enhanced activity inhibiting antitumor cell proliferation [86]. Dual inhibitors based on harmine, which selectively targets histone deacetylases (HDAC) and DNA, displayed potent antitumor efficacy against cancerous cells with minimal toxicity toward normal cells [87]. Also, to enhance its solubility, harmine was synthesized alongside an amphiphilic self-assembling polymer micelle of lactose-palmitoyl-trimethyl-chitosan (Lac-TPCS), which concurrently reduced its adverse effects [88]. Furthermore, N-trimethyl-chitosan (TMC)-coated harmine liposome (HM-lip) was fabricated, which protected against enzymatic degradation, prolonged retention time in the gastrointestinal tract, and enhanced transport across Caco-2 cell monolayers, thereby boosting oral bioavailability of harmine [89].

## Conclusions and future perspectives

This review article concerns the current information about different antitumoral activities of harmine and its synthetic derivatives in diverse preclinical models. These data highlight the strong anticancer potential of natural compounds and further justify the studies of novel lead compounds from various natural resources. Some ongoing or planned clinical trials involve harmine or its derivatives. One such trial focused on measuring how the peroral administration of the capsules containing isolated DMT and harmine compared to the combined approach of a buccal harmine tablet and an intranasal DMT spray at two dose levels. The buccal and intranasal combined technique remarkably mitigated the occurrence of gastrointestinal disorders like nausea and vomiting and further verified physical drug stability (NCT04716335).

It is expected that overcoming the bottlenecks of harmine by preparing its semisynthetic derivatives will lead to the development of more efficient and safer anticancer drugs shortly. There is a vital requirement for this, considering the continuously increasing rate of new cancer cases around the world. Moreover, combining these novel agents with conventional chemotherapeutic drugs could enhance the therapeutic responses and reduce their efficient concentrations, thereby improving patients' quality of life suffering from different malignancies.

## Data Availability

No datasets were generated or analysed during the current study.
